# The complete mitochondrial genome sequence of *Cletus rubidiventris* (Heteroptera: Coreidae)

**DOI:** 10.1080/23802359.2020.1797590

**Published:** 2020-07-29

**Authors:** Shijun Yang, Rong Hou, Kongju Wu, Peng Liu, Yuxiang Chen, Wanjing Yang, Peng Chen

**Affiliations:** a Chengdu Research Base of Giant Panda Breeding, Chengdu, China; b Sichuan Key Laboratory of Conservation Biology for Endangered Wildlife, Chengdu, China; c Sichuan Academy of Giant Panda, Chengdu, China; d College of Life Science, China West Normal University, Nanchong, China

**Keywords:** *Cletus rubidiventris*, mitochondrial genome, phylogenetic analysis

## Abstract

*Cletus rubidiventris* is a crop pest, especially for rice. This study first reported the complete mitochondrial genome of this species. The total length of mitochondrial genome is 15,590 bp and including 13 PCGs, 22 tRNA genes, and 2 rRNA genes, with 31.8% T, 15.8% C, 41.6% A, and 10.8% G. The overall GC content of the genome is 27%. The mitochondrial genome order, nucleotide composition, and codon usage pattern is similar to *C. punctiger*. The phylogenetic tree shows that *C. rubidiventris* belong to the Coreidae.

*Cletus rubidiventris*, belonging to the Coreidae, is a pest of rice and feeding on young panicle. It often causes a decline of rice yield. So far, the molecular studies on the mitochondrial total sequence of the genus *Cletus* have only completed the determination of mtDNA sequence in *C. punctiger* (Zhang et al. 2019). Herein, the complete mitochondrial genome of *C. rubidiventris*, its genetic structures and the phylogenetic relationship were first discussed.

The specimens of *C. rubidiventris* were collected from campus of Guizhou Normal University (26°22′50.30″N, 106°38′11.72″E) and stored at Chengdu Research Base of Giant Panda Breeding (voucher number: CPC201913). The complete mitochondrial genome was sequenced using Illumina HiseqXten and was assembled using SOAPdenovo2 (Luo et al. 2012). *Mictis tenebrosa* (Accession number: NC_042811) was used as reference genome (Liu et al. 2019). The mitogenome sequence was aligned and analyzed by MEGA 5.0 (Tamura et al. 2011). Phylogenetic analysis by maximum likelihood (ML) was performed using RAxML 8.2.10 (Stamatakis 2014) under the GTRGAMMA model, using 1000 bootstrap replicates.

The complete mitochondrial genome of *C. rubidiventris* is 15,590 bp in length, it is contained 37 typical mitochondrial genes (13 PCGs, 22 tRNAs, and 2 rRNAs). The total base composition of the mitochondrial genome is 31.8% T, 15.8% C, 41.6% A, and 10.8% G, and with 27% GC content, which is similar to *C. punctiger*. Five PCGs (COII, ND1, ND2, ND3 and ND4L) starting with ATT initiation codon, three PCGs (COIII, ND4, ND5 and ATP6) starting with the ATG codon, two PCGs (CYTB and ND6) with ATA codon, COI and ATP8 with TTG and ATC codon, respectively. Nine PCGs used TAA as stop codon, ND4 and ND5 used TAG as stop codon, but CYTB and ND1 used TA- as in complete stop codon. Among the 13 protein-coding genes, the longest gene was ND5 (1713 bp), while the shortest gene was ATP8 (160 bp).

The ML phylogenetic tree was constructed with the whole mitochondrial genome of Coreidae and set *Tachyta nana* as outgroup (Figure 1). Nodes with >70 bootstrap (BS; Hillis and Bull 1993) were considered to the strongly supported. The result of phylogeny tree shows *C. rubidiventris* nested with Coreidae and it is sister to *Cloresmus pulchellus* with highly supports (BS = 99). This is the first time to reveal the complete mitochondrial genome of *C. rubidiventris*. This not only can provide important data for the phylogenetic evolution research and genetic identification, but also contribute to the conservation of ecology.

**Figure 1.**
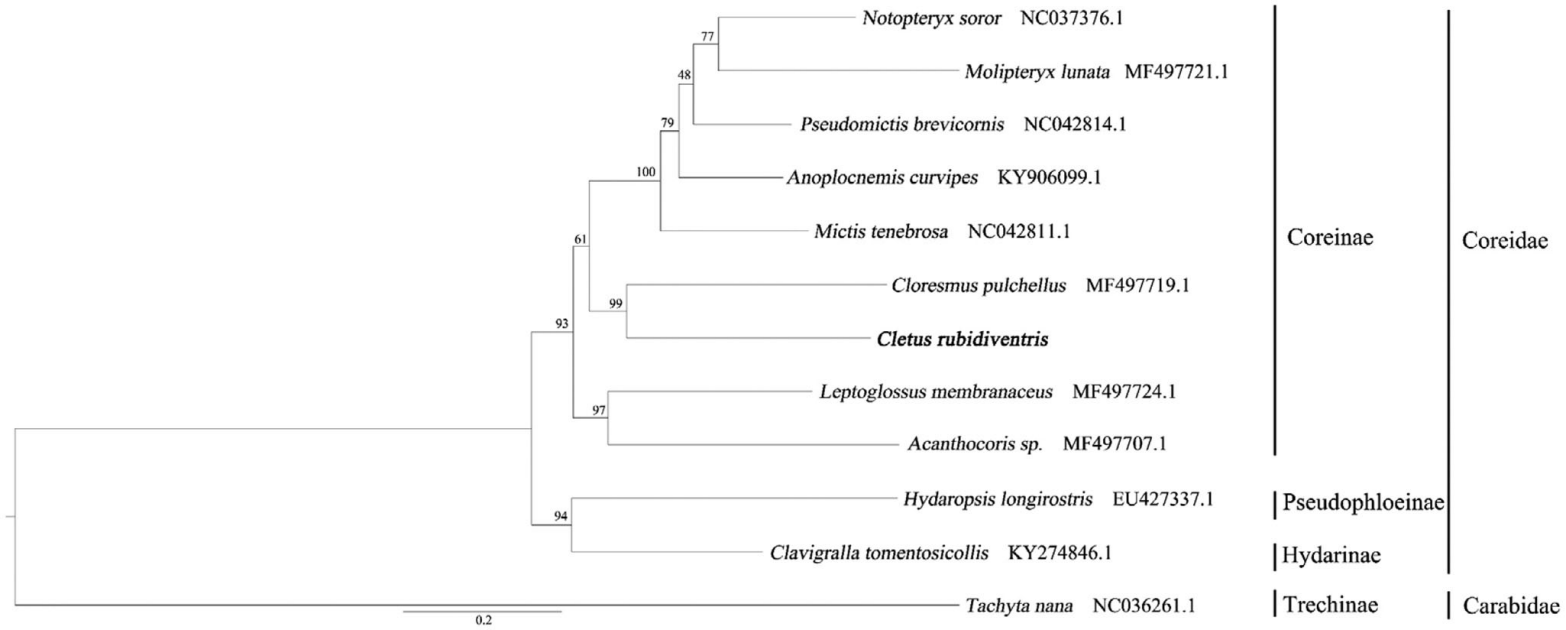
Maximum likelihood phylogenetic tree of *Cletus rubidiventris* constructed with 12 species based on mitochondrial genome. The phylogenetic tree includes the maximum likelihood bootstrap (BS) values of the nodes.

## Data Availability

The data that support the findings of this study are openly available in GenBank of NCBI at https://www.ncbi.nlm.nih.gov, reference number MT702884.
